# Carbon tetrachloride does not promote hepatic fibrosis in ob/ob mice via downregulation of lipocalin-2 protein

**DOI:** 10.1016/j.redox.2025.103506

**Published:** 2025-01-16

**Authors:** Hyun Joo Shin, Kyung Eun Kim, Hyeong Seok An, Eun Ae Jeong, Jiwon Oh, Yundong Sun, Dong-Ju Park, Jaewoong Lee, Jinsung Yang, Gu Seob Roh

**Affiliations:** a Department of Anatomy and Convergence Medical Science, College of Medicine, Metabolic Dysfunction Liver Disease Research Center, Institute of Medical Science, Gyeongsang National University, Jinju, 52727, Republic of Korea; b Department of Biochemistry, College of Medicine, Institute of Medical Science, Gyeongsang National University, Jinju, 52727, Republic of Korea

**Keywords:** Carbon tetrachloride, Hepatic fibrosis, Lipocalin-2, Ob/ob mouse

## Abstract

Although leptin-deficient ob/ob mice have been investigated to determine whether hepatic steatosis promotes susceptibility to hepatotoxic insults, carbon tetrachloride (CCl_4_)-induced hepatic fibrosis in ob/ob mice remains largely unknown. In this study, we evaluate the pathogenic mechanisms of hepatic fibrosis in CCl_4_-treated wild-type (WT) and ob/ob mice and analyze some parameters related to lipogenesis, inflammation, fibrosis, oxidative stress, apoptosis, and autophagy. CCl_4_ treatment attenuated liver weight and lipogenesis in ob/ob mice. Increased hepatic fibrosis-related proteins were reduced in CCl_4_-treated ob/ob mice compared with CCl_4_-treated WT mice. Specifically, the expression of lipocalin-2 (LCN2) was markedly reduced in CCl_4_-treated ob/ob mice versus CCl_4_-treated WT mice. Compared with CCl_4_-treated WT mice, CCl_4_-treated ob/ob mice had reduced expression of neutrophil-related inflammatory genes and proteins. Hepatic heme oxygenase-1 protein was reduced in CCl_4_-treated ob/ob mice compared with CCl_4_-treated WT mice. However, CCl_4_ did not promote hepatic apoptosis in ob/ob mice. Therefore, these findings highlight LCN2 as a key signaling factor in CCl_4_-induced hepatic fibrosis.

## 1 Introduction

The development of novel therapies for metabolic dysfunction-associated steatotic liver disease (MASLD) has garnered substantial attention owing to the increasing prevalence of obesity and type 2 diabetes worldwide [1]. Simple steatosis, in which lipid droplets accumulate in hepatocytes, progresses to metabolic dysfunction-associated steatohepatitis (MASH) accompanied by fibrosis and inflammatory cell infiltration, and finally, liver cirrhosis with severe fibrosis [2,3]. Fatty liver also increases the risk of complications and mortality from liver transplantation and partial hepatectomy [4,5].

A rodent model of carbon tetrachloride (CCl_4_)–induced hepatic fibrosis, wherein free radicals induce liver damage through lipid peroxidation, is the most extensively studied hepatic fibrosis model [6]. Leptin is known to induce the development of hepatic fibrosis via the activation of hepatic stellate cells (HSCs) [7]. Furthermore, leptin administration accelerates CCl_4_-induced hepatic fibrosis but reduces fibrosis in leptin-deficient ob/ob mice [8]. These mechanisms, however, remain to be completely delineated.

Ob/ob mice are used as models of fatty liver wherein lipid droplets accumulate due to increased lipogenesis within hepatocytes [9,10]. These models have been used to test hypotheses that hepatic steatosis promotes susceptibility to hepatotoxic insults. Particularly, leptin deficiency causes impaired liver regeneration in ob/ob mice, and altered cytokines expression is associated with impaired hepatic fibrosis in response to CCl_4_ treatment [11]. However, Potter et al. suggested that through regulating transforming growth factor-β1 (TGF-β1), leptin deficiency results in a decrease, but not complete elimination of hepatic fibrosis induced by CCl_4_ treatment and *Schistosoma mansoni* infection [12]. In our previous study, we demonstrated that lipocalin-2 (LCN2) promotes hepatic fibrosis in high-fat diet (HFD)-fed ob/ob mice by activating HSCs [13]. Therefore, leptin may be a potentiating, but not an essential factor in the development of hepatic fibrosis.

In the present study, studying the potential role of LCN2 in CCl_4_-treated ob/ob mice can provide valuable insights into the pathophysiology of fatty liver and subsequent progression to hepatic fibrosis and can help to identify potential therapeutic targets.

## 2 Materials and methods

### 2.1 Experimental animal models

Male C57BL/6 J wild-type (WT) and leptin-deficient ob/ob mice were purchased from Central Laboratory Animal, Inc. (Seoul, Republic of Korea). LCN2 knockout (KO) mice were purchased from The Jackson Laboratory (Bar Harbor, ME, USA). The absence of LCN2 was confirmed by PCR analysis of genomic DNA. For a CCl_4_ (Sigma-Aldrich, Inc., St Louis, MO, USA)-induced liver injury model, male WT and ob/ob mice received an intraperitoneal injection of CCl_4_ (1 ml/kg) in olive oil (Sigma-Aldrich) twice a week for 4 weeks starting at 10 weeks of age. All animal procedures adhered to the National Institutes of Health Guidelines for the Use of Laboratory Animals and the study protocol was approved by the University Animal Care Committee for Animal Research of Gyeongsang National University (GNU-170116-M0022). Mice were individually housed under an alternating 12 h light/dark cycle.

### 2.2 Measurement of serum metabolic parameters

Mice (*n* = 10 mice) were intraperitoneally anesthetized with 1.25 % avertin (Sigma-Aldrich). From the left ventricle, blood samples were taken and centrifuged. Serum aspartate aminotransferase (AST), alanine aminotransferase (ALT), total cholesterol, and glucose levels were measured at the SCL Healthcare (Yongin, Republic of Korea). Serum leptin, insulin, and LCN2 concentrations were measured using mouse leptin (R&D Systems, Minneapolis, MN, USA), insulin (Shibayagi Co., Gunma, Japan), and LCN2 (R&D Systems) enzyme-linked immunosorbent assay (ELISA) kits according to the manufacturer's protocols.

### 2.3 Tissue collection and histological analysis

After removing the blood, the extracted liver (*n* = 3–4 mice) was fixed in 4 % paraformaldehyde for 12 h at 4 °C and embedded in frozen and paraffin, and cut into 5-μm sections. Nile Red (Sigma-Aldrich) staining for hepatic TG and hematoxylin and eosin (H&E, Abcam, Cambridge, MA, USA) staining were performed on frozen and paraffin-embedded liver sections, respectively. The percentage of Nile Red-positive area (500 × 500 μm^2^) in three sections was determined using ImageJ software (Version 1.52a, NIH, Bethesda, MD, USA). Sections were visualized using an FV3000 microscope (Olympus, Tokyo, Japan). Hepatic fibrosis was identified on Sirius Red (Sigma-Aldrich) and Masson trichrome (MT, Polysciences Inc., PA, USA) staining according to the manufacturer's protocols. To determine iron accumulation in hepatocytes, we performed Perls Prussian blue staining (Iron Stain Kit, Abcam). The sections were visualized using a microscope slide scanner (Motic, Hong Kong, China). Sirius Red- and Masson trichrome-positive areas in 15 fields (magnification x100) from 3 mice/group were quantified using ImageJ software (Version 1.52a).

### 2.4 Atomic force microscopy (AFM)

Measurements on paraffin liver sections (5 μm) were performed. AFM height images were obtained using an AFM (Nanowizard 4XP, JPK, PeakForce QNM mode, Berlin, Germany). The height images were recorded with ScanAsyst-Fluid (Bruker, diameter 20 μm). The cantilevers were calibrated using the thermal noise method, oscillating at a frequency of 0.1 kHz and an amplitude of 1 μm. The AFM is combined with an inverted optical microscope (Axio observer 3, Zeiss). The bright field images were obtained with a 40× objective. FD curves were analyzed with JPK data processing software (v8.0.154). Young's modulus values were extracted by fitting Hertz's model. The AFM images were obtained from 10 μm × 10 μm. The samples are from each mouse liver section**,** and 3 different areas were recorded.

### 2.5 MASLD activity score measurement

The activity score of MASLD was defined as an unweighted sum of scores for liver steatosis (0–3), lobular inflammation (0–3), and hepatocyte ballooning (0–2) [14].

### 2.6 *Hepatic* triglyceride *(TG) colorimetric assay*

Frozen livers were homogenized and centrifuged, and the supernatants were used to determine TG levels using a TG colorimetric assay kit (Cayman Chemical Company, Ann Arbor, MI, USA).

### 2.7 RNA isolation and quantitative real-time PCR (RT-PCR)

Total RNA was extracted from livers (*n* = 6 mice), using the TRIzol reagent (Invitrogen, Waltham, MA, USA) and reverse-transcribed using the RevertAid First Strand cDNA Synthesis Kits (Thermo Fisher Scientific). Quantitative RT-PCR was performed using the LightCycler 480 Instrument II (Roche Diagnostics GmbH). PCR amplifications were treated with iQ™ SYBR Green Supermix (Bio-Rad Laboratories, Hercules, CA, USA) with specific primers (Supplementary Table 1). Relative quantifications were calculated using the ΔΔCt formula. Relative mRNA expression was expressed as a fold-change relative to a calibrator sample. All samples were run in duplicate, and average values were calculated.

### 2.8 Tissue fractionation and Western blot analysis

For total protein extraction (*n* = 3–4 mice), frozen livers and epididymal fat pads were homogenized in T-PER lysis buffer (Thermo Fisher Scientific) with a protease and phosphatase inhibitor cocktail (Thermo Fisher Scientific). For cytosolic and nuclear fractions, we used the NE-PER Nuclear and Cytoplasmic Extraction Kit (Pierce, Rockford, IL, USA). Protein concentration was measured by a BCA assay (Thermo Fisher Scientific). The primary antibodies used are shown in Supplementary Table 2 β-actin and p84 were used as loading controls to normalize protein levels in total and nuclear fractions, respectively. Protein bands were detected using enhanced chemiluminescence substrates (Thermo Fisher Scientific), and chemiluminescence was analyzed using a LAS-4000 instrument (Fujifilm, Tokyo, Japan). The Multi-Gauge V 3.0 image analysis program (Fujifilm) was used for densitometry analysis.

### 2.9 Cytokine and chemokine array

Pooled liver lysates (*n* = 4 mice) from WT or ob/ob mice treated with oil or CCl_4_ were analyzed using the Proteome Profiler Mouse Cytokine Array Kit (R&D systems) to detect 40 different cytokines and chemokines according to the manufacturer's protocol.

### 2.10 Immunohistochemistry

Deparaffinized liver sections were placed in 0.3 % H_2_O_2_ for 30 min, rinsed, and incubated in a blocking normal serum for 1 h at room temperature. After rinsing, the sections were incubated with a primary antibody (Supplementary Table 2) at 4 °C overnight and thereafter with a secondary biotinylated antibody at room temperature for 1 h. After rinsing, the sections were incubated with an avidin-biotin-peroxidase complex solution (Vector Laboratories, Burlingame, CA, USA) and developed using a 0.05 % diaminobenzidine substrate kit (Vector Laboratories). Slides were visualized using a microscope slide scanner (Motic).

### 2.11 Hepatic hydroxyproline assay

Hepatic hydroxyproline levels in frozen livers (*n* = 4–5 mice) were measured using a mouse hydroxyproline assay kit (Cell Biolabs, Inc., San Diego, CA, USA).

### 2.12 Immunofluorescence

Deparaffinized liver sections were incubated with 5 % serum for 1 h at room temperature followed by incubation with a primary antibody and the corresponding Alexa Fluor 594- or 680- conjugated secondary antibody according to Supplementary Table 2. Additionally, we performed double immunofluorescence staining for glutamine synthetase (GS) and terminal deoxynucleotidyl transferase dUTP nick end labeling (TUNEL) to measure the percentage of GS-positive apoptotic adipocytes using an In-Situ Cell Death Detection Kit (Roche Molecular Diagnostics, Mannheim, Germany) according to the manufacturer's protocol. The nuclei were counterstained with 4′,6-diamidino-2-phenylindole (DAPI, Invitrogen). Mounted slides were visualized using an FV3000 microscope (Olympus). GS- and TUNEL-positive areas in 15 fields (magnification × 100) from 3 mice per group were quantified using ImageJ software (Version 1.52a).

### 2.13 Transmission electron microscopy (TEM)

Mice (*n* = 2 mice) were anesthetized and perfused with both 2 % PFA and 2 % glutaraldehyde in 0.1 M PBS. The liver specimen was fixed using the same fixative solution for 18 h at 4 °C. Then, the sections were rinsed with 0.1 M PBS and osmicated with 1 % osmium tetroxide for 90 min. The sections were then dehydrated using graded alcohol concentrations, infiltrated with propylene oxide for 10 min, and embedded using the Poly/Bed 812 Embedding Kit (Polysciences Inc., Warrington, FL, USA) for 18 h. The sections were cut at 60 nm with a diamond knife and stained with 5 % uranyl acetate for 10 min and 1 % lead citrate for 5 min. Images were obtained using a transmission electron microscope (JEM-1011, JEOL Ltd, Tokyo, Japan) at an 80-kV accelerating voltage and were photographed with a digital CCD camera (EMSIS GmbH, Muenster, Germany).

### 2.14 Statistical analysis

Statistical analyses were performed using PRISM 9.0 (GraphPad Software Inc., San Diego, CA, USA). Group differences were determined using one-way and two-way analysis of variance (ANOVA) followed by Tukey's *post hoc* tests. Data are expressed as mean ± standard error of the mean (S.E.M.). A *p*-value <0.05 was considered statistically significant.

## 3 Results

### 3.1 CCl_4_ treatment does not aggravate liver injury in ob/ob mice

**Fig. 1:**
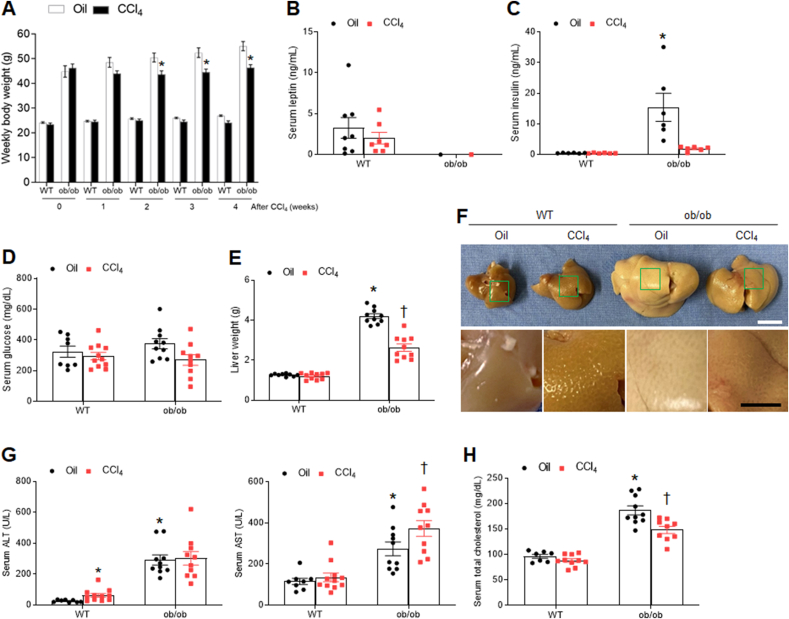
**Effects of CCl**_**4**_**on metabolic parameters in WT and ob/ob mice. (A)** Weekly body weight changes over 4 weeks after CCl_4_ treatment. Levels of serum **(B)** leptin, **(C)** insulin, **(D)** and glucose, and **(E)** liver weight. **(F)** Representative macroscopic pictures of livers. Scale bar, 1 cm. **(G)** Serum ALT and AST and **(H)** total cholesterol concentrations. Significance is determined using two-way ANOVA. ∗P < 0.05 vs. oil-treated WT. †P < 0.05 vs. CCl_4_-treated WT.

To assess the impact of CCl_**4**_ treatment on weight change in WT and ob/ob mice, we monitored their body weight for 4 weeks after CCl_4_ injection. WT mice showed no significant weight loss or change in serum leptin levels, whereas the body weight of ob/ob mice decreased 2 weeks post-injection (Fig. 1A and B). Although ob/ob mice had higher serum insulin levels than WT mice, their insulin level was significantly attenuated by CCl_**4**_ (Fig. 1C). However, there was no significant change in non-fasting serum glucose levels in ob/ob mice with oil or CCl_**4**_ (Fig. 1D). Paralleling the decrease in insulin levels, the liver weight of ob/ob mice also decreased following CCl_**4**_ injection (Fig. 1E). When observing the liver surface, CCl_**4**_-treated WT mice had not smooth surface, whereas ob/ob mice had yellowish fatty and smooth liver surface (Fig. 1F). Although serum ALT levels were higher in CCl_**4**_-treated WT mice than in oil-treated WT mice, ob/ob mice had considerably higher serum ALT and AST levels than CCl_**4**_-treated WT mice (Fig. 1G). Similar to the change in liver weight, increased serum total cholesterol levels in ob/ob mice were also significantly reduced by CCl_**4**_ injection (Fig. 1H). These results suggest that CCl_**4**_ injection do not promote liver damage in ob/ob mice.

### 3.2 CCl_4_ treatment attenuates the accumulation of hepatic lipids in ob/ob mice

**Fig. 2:**
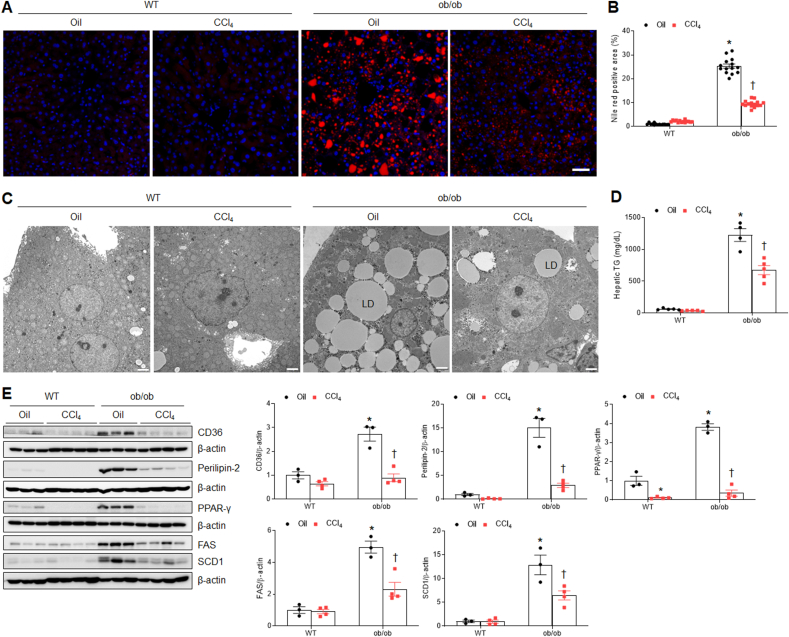
**Effects of CCl**_**4**_**on hepatic lipid accumulation in WT and ob/ob mice. (A)** Representative Nile Red with DAPI staining of liver sections. Scale bar, 50 μm. **(B)** Nile Red-positive area (%). **(C)** Electron micrographs of hepatocytes. LD indicates lipid droplet. Scale bar, 2㎛. **(D)** Hepatic TG levels. **(E)** Western blot analysis and quantification of CD36, perilipin-2, PPAR-γ, FAS, and SCD1 proteins in liver lysates. β-actin is used as a loading control. Significance is determined using two-way ANOVA. ∗P < 0.05 vs. oil-treated WT. †P < 0.05 vs. CCl_4_-treated WT.

We assessed the effects of CCl_4_ treatment on fatty acid uptake and *de novo* lipogenesis in ob/ob mice because it reduced the liver weight and serum total cholesterol levels. In addition to reduced hepatic TG levels, Nile Red staining and electron microscopy showed that the increased lipid accumulation within hepatocytes in ob/ob mice was substantially reduced by CCl_**4**_ (Fig. 2A–D). To determine whether CCl_4_ affects fatty acid uptake and *de novo* lipogenesis in ob/ob mice, cluster of differentiation 36 (CD36), perilipin-2, peroxisome proliferator-activated receptor-γ (PPAR-γ), fatty acid synthase (FAS), and stearoyl-CoA desaturase 1 (SCD1) proteins levels were evaluated using Western blot analysis. We found that although the expressions of these proteins were significantly increased in oil-treated ob/ob mice, their protein levels were reduced by CCl_**4**_ (Fig. 2E). These findings indicate that gradual weight loss during 4 weeks after CCl_4_ injection may have influenced the reduction in hepatic lipid accumulation in ob/ob mice.

### 3.3 CCl_4_ treatment attenuates hepatic fibrosis in ob/ob mice

**Fig. 3:**
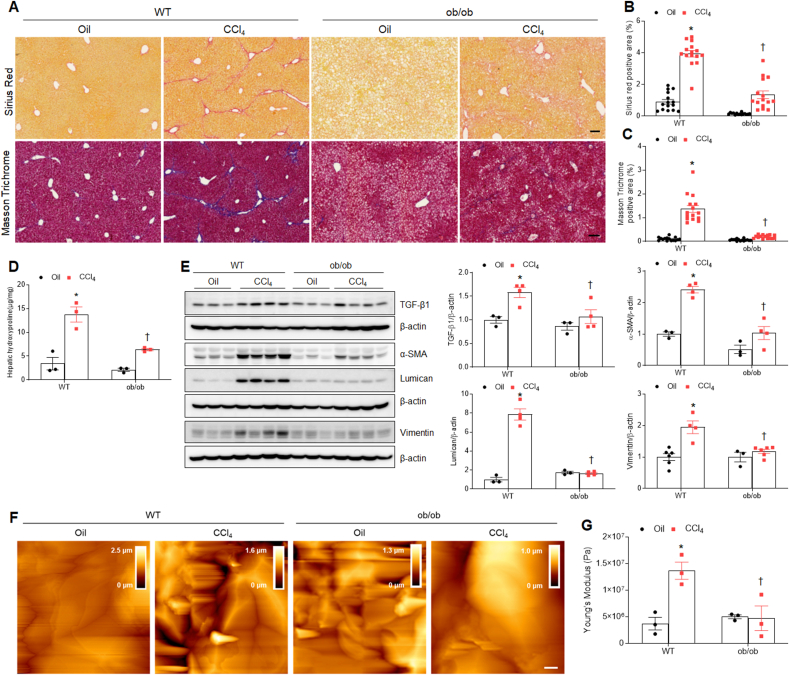
**Effects of CCl**_**4**_**on hepatic fibrosis in WT and ob/ob mice. (A)** Representative images of Sirius Red and Masson trichrome staining of liver sections. Scale bar, 60 μm. **(B–C)** Quantification of Sirius Red (B) and Masson trichrome (C) staining. **(D)** Hepatic hydroxyproline concentration. **(E)** Western blot analysis and quantification of TGF-β1, α-SMA, lumican, and vimentin proteins in liver lysates. β-actin is used as a loading control. **(F)** Height images in liver sections. Scale bar, 1 μm. **(G)** Young's modulus (%) was obtained from force-distance curves using AFM measurements. Significance is determined by two-way ANOVA. ∗P < 0.05 vs. oil-treated WT. †P < 0.05 vs. CCl_4_-treated WT.

Given that hepatic steatosis promoted vulnerability to hepatotoxic insults, we assessed the effect of CCl_4_ treatment on hepatic fibrosis in ob/ob mice. In addition to decreased hepatic hydroxyproline levels, Sirius Red and MT staining revealed that CCl_4_-treated ob/ob mice had less hepatic fibrosis than CCl_4_-treated WT mice (Fig. 3A–D). As shown in Fig. 3E, increased hepatic TGF-β1, α-smooth muscle actin (α-SMA), lumican, and vimentin proteins in CCl_4_-treated WT mice were significantly attenuated in CCl_4_-treated ob/ob mice. Furthermore, AFM revealed that CCl_4_-treated WT mice showed increased stiffness in the liver sections, whereas CCl_4_-treated ob/ob mice had relatively less stiffness (Fig. 3F and G). These results suggest that CCl_4_ treatment does not promote hepatic fibrosis in ob/ob mice with fatty liver.

### 3.4 Hepatic LCN2 protein is reduced in CCl_4_-treated ob/ob mice

**Fig. 4:**
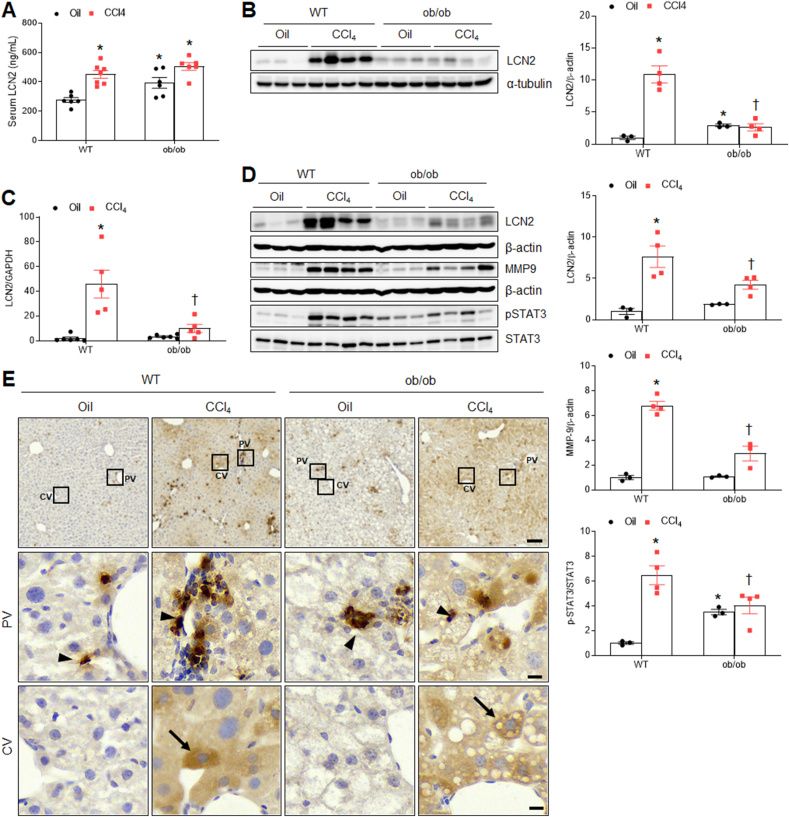
**Effects of CCl**_**4**_**on hepatic LCN2 protein expression in WT and ob/ob mice. (A)** Serum LCN2 levels. **(B)** Western blot analysis and quantification of LCN2 protein in epididymal fat pad lysates. α-Tubulin is used as a loading control. **(C)** LCN2 mRNA in liver lysates quantified using qPCR. **(D)** Western blot analysis and quantification of LCN2, MMP9, pSTAT3, and STAT3 proteins in liver lysates. β-actin is used as a loading control. **(E)** Representative LCN2 immunostaining of liver sections. PV and CV indicate portal vein and central vein, respectively. The Arrowhead and arrow indicate LCN2-positive Kupffer cells and hepatocytes, respectively. Scale bar, 100 μm (upper panel), 10 μm (middle and lower panels). Significance is determined using two-way ANOVA. ∗P < 0.05 vs. oil-treated WT. †P < 0.05 vs. CCl_4_-treated WT.

Given that LCN2 promoted hepatic fibrosis, we measured LCN2 protein levels in the serum, epididymal fat pads, and liver. Notably, we found that serum LCN2 levels increased in all mice groups except in oil-treated WT mice (Fig. 4A). Similar to LCN2 levels in epididymal fat pads, hepatic LCN2 mRNA and protein levels were higher in CCl_4_-treated WT mice than in CCl_4_-treated ob/ob mice (Fig. 4B–D). Increased matrix metalloproteinase 9 (MMP9) and phosphorylated signal transducer and activator of transcription 3 (pSTAT3) in CCl_4_-treated WT mice were also significantly reduced in CCl_4_-treated ob/ob mice (Fig. 4D). To identify the target cells responsible for LCN2-mediated fibrosis in CCl_4_-treated WT and ob/ob mice, we performed immunohistochemistry. Compared with oil-treated WT and ob/ob mice, numerous LCN2-positive cells were observed in both Kupffer cells and hepatocytes in CCl_4_-treated WT and ob/ob mice (Fig. 4E). To additionally validate the effect of LCN2 on hepatic fibrosis, we assessed hepatic α-SMA expression in CCl_4_-treated WT, ob/ob, and LCN2KO mice. Among the three mouse groups, ob/ob mice had the lowest hepatic α-SMA protein compared to the other two groups (Supplementary Fig. 1A). LCN2 deletion also attenuated hepatic α-SMA protein expression compared to CCl_4_-treated WT mice. On the other hand, to determine whether LCN2 affects iron accumulation in CCl_4_-induced hepatic fibrosis, we performed Perls Prussian blue iron staining. Notably, we found that compared to CCl_4_-treated WT mice, iron-stained hepatocytes were reduced in CCl_4_-treated ob/ob mice (Supplementary Fig. 1B). These findings suggest that LCN2-mediated signaling may be associated with CCl_4_-induced hepatic fibrosis.

### 3.5 Hepatic inflammation is reduced in CCl_4_-treated ob/ob mice

**Fig. 5:**
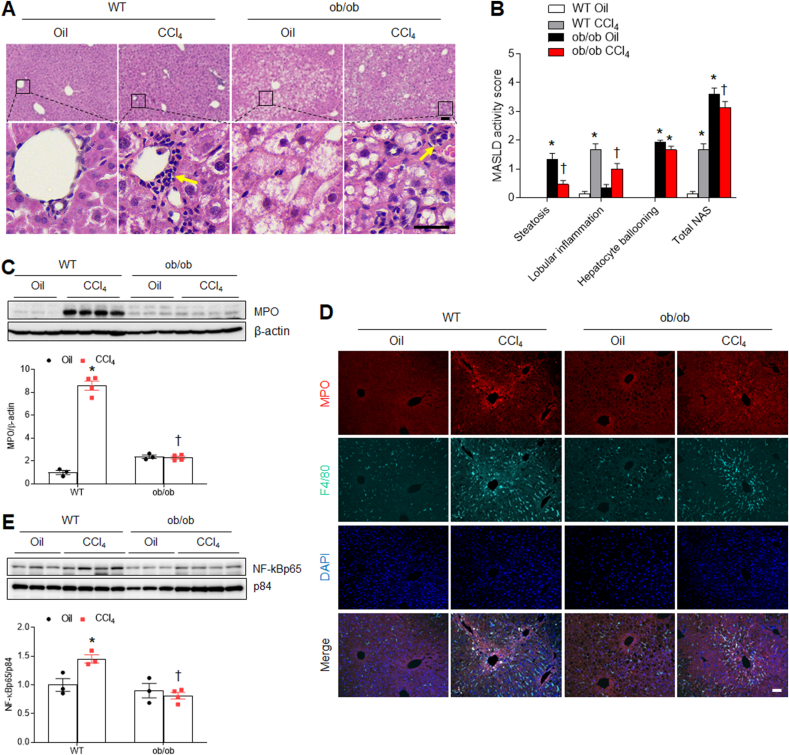
**Effects of CCl**_**4**_**on hepatic inflammation in WT and ob/ob mice. (A)** Representative images of H&E staining of liver sections. Scale bars, 60 μm (upper panel), 30 μm (lower panel). Yellow arrows indicate neutrophils. **(B)** MASLD activity score. **(C)** Western blot analysis and quantification of MPO protein in liver lysates. β-actin is used as a loading control. **(D)** Representative MPO and F4/80 immuno-staining of liver sections. Nuclei were counterstained with DAPI. Scale bar, 100 μm. **(E)** Western blot analysis and quantification of nuclear NF-kBp65 protein in liver lysates. p84 is used as a loading control. Significance is determined using two-way ANOVA. ∗P < 0.05 vs. oil-treated WT. †P < 0.05 vs. CCl_4_-treated WT.

**Fig. 6:**
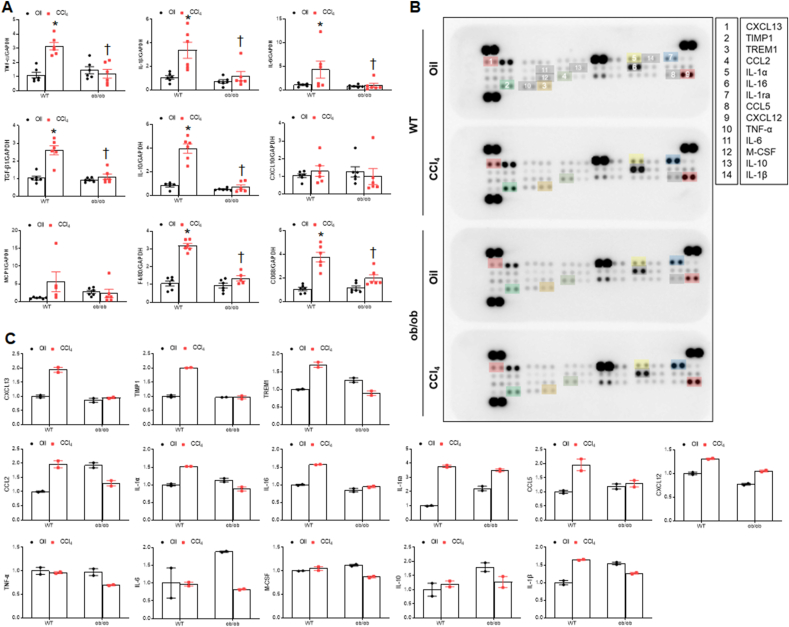
**Effects of CCl**_**4**_**on hepatic cytokine and chemokines expression in WT and ob/ob mice. (A)** Proinflammatory and anti-inflammatory cytokine and chemokine mRNA expression in liver lysates quantified using qPCR. **(B)** Cytokine and chemokine expression in liver lysates were determined using mouse cytokine arrays. **(C)** Bar graphs for each cytokine with mean pixel density. Significance is determined using two-way ANOVA. ∗P < 0.05 vs. oil-treated WT. †P < 0.05 vs. CCl_4_-treated WT.

Hepatic fibrosis is associated with inflammation with neutrophil infiltration [15]. Consistent with the MASLD activity score, CCl_**4**_ increased neutrophil infiltration around the periportal vein and hepatic myeloperoxidase (MPO) protein expression in WT mice. However, they were significantly attenuated in CCl_**4**_-treated ob/ob mice (Fig. 5A–C). Double immunofluorescence revealed that many MPO-positive neutrophils were observed with F4/80-positive Kupffer cells around the portal vein of fibrotic areas in CCl_4_-treated WT mice compared with CCl_4_-treated ob/ob mice (Fig. 5D). Furthermore, we found that increased nuclear NF-κBp65 in CCl_4_-treated WT mice was significantly reduced in CCl_4_-treated ob/ob mice (Fig. 5E). We next evaluated whether CCl_4_ affects proinflammatory and anti-inflammatory cytokine mRNA using quantitative RT-PCR. CCl_4_-induced proinflammatory mRNAs, including tumor necrosis factor (*tnf)-α, interleukin (il)-1β*, *il*-6, monocyte chemoattractant protein 1 (*mcp1)*, *f4/80*, and *cd68* and anti-inflammatory genes, such as *tgf-β1* and *il-10*, observed in the livers of WT mice were attenuated in CCl_**4**_-treated ob/ob mice, whereas C-X-C motif chemokine 10 (*cxcl10)* mRNA levels remained unchanged (Fig. 6A). In addition, using liver cytokine and chemokine array (Fig. 6B), we found that compared with CCl_**4**_-treated WT mice, tissue inhibitor matrix metalloproteinase1 (TIMP1), triggering receptor expressed on myeloid cells1 (TREM1), IL-16, IL-1α, IL-1β, chemokine ligand (CCL)2, CCL5, CXCL12, and CXCL13 were decreased in CCl_**4**_-treated ob/ob mice (Fig. 6C). However, levels of canonical cytokines, such as TNF-α, IL-6, and IL-10 showed no changes. Taken together, these findings imply that ob/ob mice may exhibit resistance to CCl_4_-induced hepatic inflammation, resulting in reduced hepatic fibrosis.

### 3.6 CCl_4_-treated ob/ob mice have more anti-oxidant effects than CCl_4_-treated WT mice

**Fig. 7:**
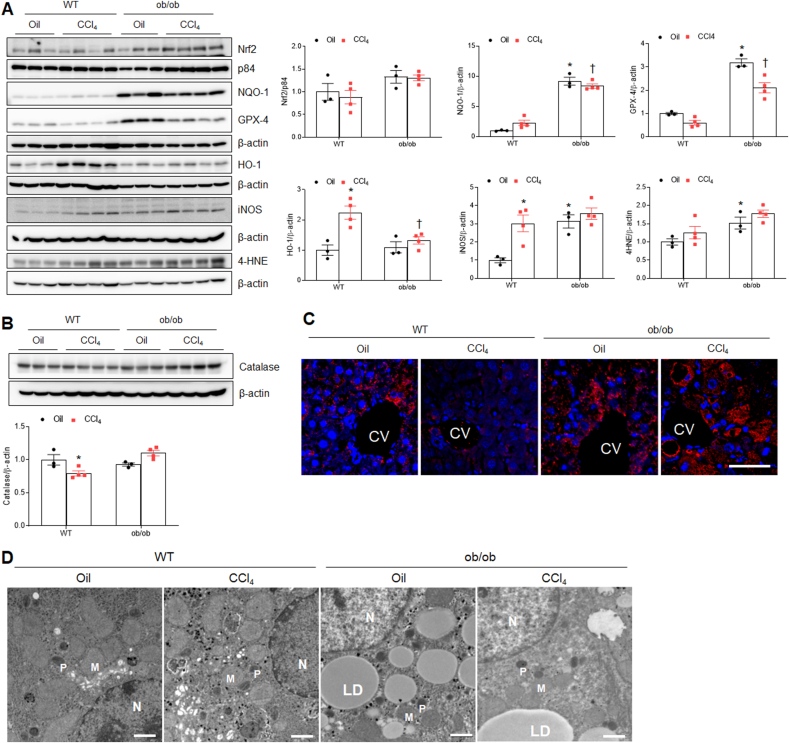
**Effects of CCl**_**4**_**on hepatic oxidative stress in WT and ob/ob mice. (A)** Western blot analysis and quantification of Nrf2, NQO-1, GPX-4, HO-1, iNOS, and 4-HNE proteins in liver lysates. Nuclear p84 and total β-actin are used as loading controls. **(B)** Western blot analysis and quantification of catalase protein in liver lysates. β-actin is used as a loading control. **(C)** Representative catalase-immunostaining of liver sections. DAPI was stained as the nucleus. CV indicates central vein. Scale bar, 50 μm. **(D)** Electron micrographs of hepatocytes. N, Nucleus; M, mitochondria; P, peroxisome; LD, lipid droplet. Scale bar, 1 μm. Significance is determined using two-way ANOVA. ∗P < 0.05 vs. oil-treated WT. †P < 0.05 vs. CCl_4_-treated WT.

Given that CCl_4_ exposure caused lipid peroxidation in the liver, we investigated whether CCl_4_ affects hepatic oxidative stress in ob/ob mice. There was increased pattern in nuclear factor erythroid 2-related factor 2 (Nrf2) in ob/ob mice compared with WT mice (Fig. 7A). We found that antioxidant protein markers NAD (P) H: quinone oxidoreductase-1 (NQO-1), and glutathione peroxidase-4 (GPX-4) protein levels were increased in ob/ob mice compared with WT mice regardless of CCl_4_ treatment (Fig. 7A). Notably, heme oxygenase-1 (HO-1) was increased in only CCl_4_-treated WT mice, whereas inducible nitric oxide synthase (iNOS) and 4-hydroxynonenal (4-HNE) protein levels were increased in all mice groups compared with oil-treated WT mice. Hepatic catalase protein, which decomposes H_2_O_2_, was significantly decreased in CCl_4_-treated WT mice. However, it was not decreased in ob/ob mice (Fig. 7B and C). Electron microscopic imaging revealed many smaller peroxisomes within hepatocytes in CCl_4_-treated WT mice than in oil-treated WT mice (Fig. 7D). Therefore, these data indicate that ob/ob mice may exhibit resistance to hepatic lipid peroxidation and reduction of peroxisome in response to CCl_4_ treatment.

### 3.7 CCl_4_ treatment does not promote hepatic apoptosis and autophagy flux in ob/ob mice

**Fig. 8:**
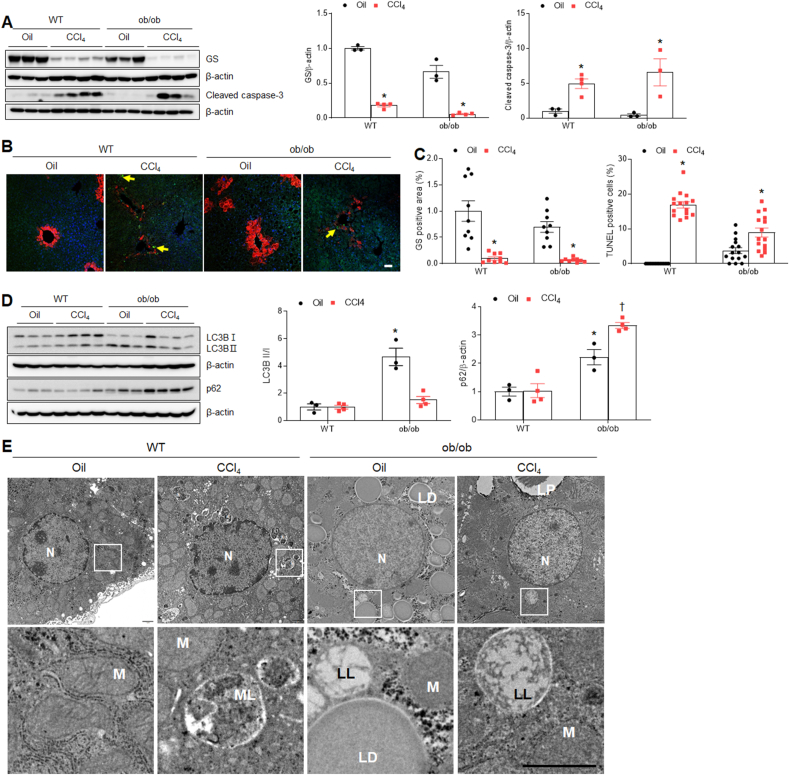
**Effects of CCl**_**4**_**on hepatic apoptosis and autophagy flux in WT and ob/ob mice. (A)** Western blot analysis and quantification of GS and cleaved caspase-3 proteins in liver lysates. β-actin is used as a loading control. **(B)** Representative GS-immuno-staining and TUNEL staining of liver sections. Yellow arrows indicate TUNEL-positive cells. DAPI was stained as the nucleus. Scale bar, 100 μm. **(C)** GS-positive area and TUNEL-positive cells. **(D)** Western blot analysis and quantification of LC3B and p62 proteins in liver lysates. β-actin is used as a loading control. **(E)** Electron micrographs of hepatocytes. N, nucleus; M, mitochondria; ML, mitolysosome; LD, lipid droplet; LL, lipolysosome. Scale bar, 1 μm. Significance is determined using two-way ANOVA. ∗P < 0.05 vs. oil-treated WT. †P < 0.05 vs. CCl_4_-treated WT.

To assess whether CCl_4_ promotes apoptosis in ob/ob mice, we evaluated the effects of CCl_4_ treatment on hepatic GS, which is a susceptible protein to oxidative damage. CCl_4_ treatment markedly attenuated hepatic GS protein in both WT and ob/ob mice (Fig. 8A). Cleaved caspase-3 protein was increased in both WT and ob/ob mice following CCl_4_ treatment (Fig. 8A). Immunofluorescent analysis showed a reduction in GS-positive hepatocytes and the presence of TUNEL-positive cells around central veins in CCl_4_-treated WT and ob/ob mice compared with oil-treated mice (Fig. 8B and C). Given that microtubule-associated protein 1A/1 B–light chain 3 (LC3) lipidation and its association with autophagosome membranes have been demonstrated to be valuable markers of autophagy, we investigated autophagic flux in CCl_4_-treated WT and ob/ob mice. As expected, a significant increase in the LC3BII/I ratio and p62 was detected in the livers of oil-treated ob/ob mice compared with oil-treated WT mice (Fig. 8D). Of note, CCl_4_ reduced the LC3BII/I ratio and increased the p62 protein level in ob/ob mice. TEM revealed increased amounts of LDs and lipolysosomes (LL, LD-loaded lysosomes) were observed in ob/ob mice compared with WT mice (Fig. 8E). Additionally, we found that mitolysosomes (MLs) around the nucleus within hepatocytes were observed in CCl_4_-treated WT mice (Fig. 8E). These findings indicate that CCl_4_ could not aggravate hepatic apoptosis and autophagic flux in ob/ob mice.

## 4 Discussion

In this study, we hypothesized that ob/ob mice are genetically predisposed to develop hepatic fibrosis due to hepatic steatosis and that CCl_4_ exposure can exacerbate liver damage by inducing inflammation, oxidative stress, apoptosis, and accelerating fibrosis. However, our findings revealed that CCl_4_ exposure could not aggravate hepatic inflammation and fibrosis in ob/ob mice via downregulating LCN2.

It has been reported that secondary insults such as methionine-choline-diet, HFD, and lipopolysaccharide accelerate hepatic fibrosis progression in ob/ob mice [13,16,17]. Hence, ob/ob mice have been investigated to determine whether hepatic steatosis promotes susceptibility to hepatotoxic insults. However, our findings suggest no significant association between serum hepatic enzyme levels and hepatic fibrosis in ob/ob mice following CCl_4_ exposure, highlighting the need to identify a diagnostic biomarker with improved accuracy of histopathological studies.

Hepatic lipid accumulation arises from both an increased uptake of FFAs and the activation of *de novo* lipogenesis [18,19]. CD36, an FFA transport protein, is upregulated in humans with MASLD [20,21]. Consistent with findings that hepatocyte-specific CD36 deficiency attenuated hepatic steatosis [21,22], we found that CCl_4_-treated ob/ob mice exhibited decreased CD36 protein. Although we did not monitor the food intake of CCl_4_-treated ob/ob mice, their body weights gradually declined following CCl_4_ injection, probably owing to CCl_4_ toxicity. Therefore, our findings suggest that in CCl_4_-treated ob/ob mice, hepatic lipogenesis may be attributed to decreased FFA uptake resulting from lowered food intake induced by CCl_4_ toxicity.

The mechanism whereby leptin loss in ob/ob mice attenuates CCl_4_-induced hepatic fibrogenesis remains to be understood. In this study, we showed that CCl_4_-treated ob/ob mice exhibited less hepatic fibrosis than CCl_4_-treated WT mice, linked to the downregulation of TGF-β1. This finding supports the notion that blocking TGF-β1 signaling pathway can prevent CCl_4_-induced hepatic fibrosis [23,24]. Similar to a fibrotic marker α-SMA, lumican, and vimentin were markedly reduced in CCl_4_-treated ob/ob mice compared with CCl_4_-treated WT mice. It has been supported that lumican is upregulated in a mouse model of MASH and CCl_4_ injection [25]. However, lumican null mice exhibited less scarring and collagen deposition. Obacunone, which has anti-inflammatory and antioxidative effects, attenuates vimentin expression in CCl_4_-treated mice [26]. Taken together, our findings suggest that CCl_4_ exposure could not aggravate hepatic fibrosis in leptin-deficient ob/ob mice.

We previously demonstrated that LCN2 promotes hepatic fibrosis in HFD-fed ob/ob mice by activating HSCs via LCN2/MMP9/STAT3-mediated signaling [13]. We observed two contrasting findings in the current study. First, LCN2/MMP9/STAT3-mediated signaling was significantly downregulated in CCl_4_-treated ob/ob mice, probably because the severe hepatic damage induced by CCl_4_ in WT mice activated HSCs more effectively than in HFD-fed ob/ob mice. Second, although serum AST and ALT levels correlated with serum LCN2 levels in ob/ob mice, serum LCN2 levels did not correlate with adipose and hepatic LCN2 levels in ob/ob mice. Notably, elevated serum LCN2 levels in ob/ob mice are associated with insulin resistance in humans, but this increase also appears to offer protection against obesity-induced insulin resistance [27,28]. Thus, we suggest that levels of circulating LCN2 as an adipokine increase in ob/ob mice with insulin resistance. Previous studies have indicated that serum LCN2 levels correlate well with serum hepatic enzymes, cholesterol, and C-reactive protein [29,30]. Given that an acute-phage protein LCN2 serves as a marker of liver damage, we propose that levels of hepatic enzymes and LCN2 may not help effectively differentiate between hepatic steatosis and fibrosis. Therefore, a novel biomarker with improved accuracy in pathological liver studies is warranted.

CCl_4_-treated hepatic injury is associated with increased activity of MPO, an index of hepatic neutrophil infiltration [31,32]. Further, chemokines, including CCL2 and CCL5, produced by macrophages, activate HSCs [33]. In this study, numerous MPO-positive cells were predominantly decreased in CCl_4_-treated ob/ob mice compared with CCl_4_-treated WT mice. Several cytokine and chemokine productions from inflammatory cells (neutrophils and Kupffer cells) were attenuated in CCl_4_-treated ob/ob mice. Although the role of neutrophils in hepatic fibrosis remains debatable, our findings indicate that cytokine-induced neutrophil chemoattractants can recruit neutrophils, thus generating a positive feedback loop that promotes hepatic fibrosis. In ob/ob mice, hepatic MPO decline may disrupt this feedback loop, leading to reduced hepatic fibrosis.

CCl_4_-induced hepatic fibrosis is also influenced by the production of excess reactive oxygen species (ROS) and lipid peroxidation [34]. In this study, we found a significant increase in NQO-1, GPX-4, iNOS, and 4-HNE protein levels in ob/ob mice compared with WT mice. However, CCl_4_-treated WT mice showed a significant increase in hepatic HO-1 and iNOS protein levels. The transcription factor Nrf2 regulates intracellular redox homeostasis as part of the antioxidant defense response [35]. Our data indicated that CCl_4_ exposure impaired the Nrf2 antioxidant defense system, worsening oxidative damage in WT mice by downregulating NQO-1 and upregulating HO-1, whereas this system remained intact in ob/ob mice with fatty liver. Interestingly, HO-1 offers both beneficial and detrimental roles depending on its metabolites, including ferrous iron [36]. In MASH, high HO-1 levels correlate with disease severity [37]. Excess ferrous iron can produce ROS through the Fenton reaction, leading to lipid peroxidation and ferroptosis [38]. Conversely, inhibiting HO-1 can worsen hepatic steatosis and fibrosis in vitro [39]. Consistent with our finding that GPX-4 protein levels decline following CCl_4_ exposure in WT mice, previous studies have confirmed this trend both in vitro and in vivo [40,41]. Our findings indicated that prolonged CCl_4_ exposure increased HO-1 expression while decreasing GPX-4 expression in hepatocytes, resulting in lipid peroxidation and subsequent ferroptosis and apoptosis. However, ob/ob mice, exhibiting antioxidant effects owing to enhanced hepatic NQO-1/GPX-4 expression, appeared to withstand the effects of sustained CCl_4_ exposure. On the other hand, ferroptosis is a nonapoptotic, iron-dependent cell death characterized by excessive accumulation of lipid peroxides and GPX4 inactivation [42]. LCN2 plays a crucial role in iron regulation and transport in the liver [43]. Given that ferroptosis was the main cause of liver damage driven by iron accumulation, we found that the induction of iron-stained hepatocytes in the liver section of CCl_4_-treated WT mice was reduced in CCl_4_-treated ob/ob mice. This finding supports that increased hepatic LCN2 and decreased GPX4 protein expressions in CCl_4_-treated WT mice are reversed in CCl_4_-treated ob/ob mice, suggesting that CCl_4_-induced ferroptosis may be linked to LCN2-mediated iron accumulation. Catalase, located primarily in peroxisomes, is an antioxidant involved in the β-oxidation of very long-chain fatty acids [44]. Under normal redox conditions, catalase protects against H_2_O_2_-induced damage [45]. In the presence of catalase inhibitors, the number of peroxisomes decreases while H_2_O_2_ levels increase [46]. In this study, we found that catalase protein was reduced upon CCl_4_ exposure, with lower catalase activity in CCl_4_-treated WT mice than in CCl_4_-treated ob/ob mice, indicating that catalase protects against peroxisome degradation during lipid accumulation in ob/ob mice. Although ob/ob mice exhibited resistance to oxidative stress, pericentral GS-positive hepatocytes were not sheltered against CCl_4_-induced apoptosis in ob/ob mice. Our findings, therefore, suggest that although ob/ob mice may be less susceptible to oxidative stress–induced hepatic fibrosis, they are not entirely protected against CCl_4_-induced apoptosis.

An elevated LC3BⅡ/Ⅰ ratio indicates that enhanced autophagic flux reduces lipid accumulation in hepatocytes, thereby mitigating lipotoxicity [47,48]. We hypothesized that impaired autophagic flux in ob/ob mice could exacerbate lipotoxicity following CCl_4_ treatment. However, our findings showed that although CCl_4_ treatment resulted in a decreased LC3BⅡ/Ⅰ ratio and an increased level of p62 protein in ob/ob mice, mitophagy was evident in CCl_4_-treated WT mice without changes in their LC3BⅡ/Ⅰ ratio or p62 levels. This data suggests that autophagic flux is crucial for protecting ob/ob mice from hepatic lipid accumulation with CCl_4_-induced lipid peroxidation having minimal effects on autophagic flux in ob/ob mice.

Clinically, although fatty liver increases the risk of complications and mortality from liver transplantation and partial hepatectomy, its mechanism has complex and context-dependent effects on hepatic fibrosis. The overall impact depends on the balance between pro-inflammatory and anti-oxidative stress, as well as interactions with other cell types in the liver microenvironment. For translating our findings to clinical practice, clinical trials are needed to evaluate the efficacy and safety of targeting LCN2 or enhancing antioxidant defenses in MASLD patients. Combination therapies targeting both LCN2 and antioxidant pathways, along with lifestyle modifications, may provide more comprehensive treatment strategies for MASLD.

It has been known that LCN2 has demonstrated the ability to distinguish between various stages of liver disease. In steatotic conditions, serum LCN2 levels are significantly increased in patients with MASLD, correlating with the degree of steatosis. LCN2 expression is markedly elevated in liver fibrosis models, suggesting its role in fibrogenesis. However, there are some limitations in clinical applications. First, there is a lack of specificity: LCN2 is involved in various pathological conditions beyond liver diseases, including renal damage, brain injury, and cancer. This broad involvement may limit its specificity as a liver-specific biomarker. Second, LCN2 expression can vary depending on the specific liver condition and its stage. Finally, while LCN2 shows high sensitivity and specificity for conditions like MASH, it may still require complementary tests for a definitive diagnosis, especially in differentiating between various stages of MASLD.

In conclusion, we demonstrate that CCl_**4**_-induced hepatic fibrosis was closely associated with hepatic inflammation, oxidative stress, apoptosis, and autophagy, but these adverse effects were less severe in leptin-deficient ob/ob mice than in WT mice through downregulating LCN2-related signaling. So, we highlight that LCN2 can emerge as a potential biomarker for differentiating stages of liver disease.

## CRediT authorship contribution statement

**Hyun Joo Shin:** Writing – original draft, Visualization, Methodology, Investigation, Funding acquisition, Formal analysis, Data curation, Conceptualization. **Kyung Eun Kim:** Writing – original draft, Methodology, Investigation, Funding acquisition, Formal analysis, Data curation, Conceptualization. **Hyeong Seok An:** Investigation, Formal analysis. **Eun Ae Jeong:** Investigation, Formal analysis. **Jiwon Oh:** Investigation, Formal analysis. **Yundong Sun:** Investigation, Formal analysis. **Dong-Ju Park:** Writing – review & editing, Formal analysis. **Jaewoong Lee:** Writing – review & editing, Formal analysis. **Jinsung Yang:** Writing – review & editing, Methodology. **Gu Seob Roh:** Writing – review & editing, Writing – original draft, Supervision, Project administration, Methodology, Funding acquisition, Formal analysis, Conceptualization.

## Funding

This study was supported by the Basic Science Research Program through the National Research Foundation (NRF) of Korea (RS-2023-00219399 to G.S.R., 2022R1I1A1A01067302 to H.J.S., and 2022R1I1A1A01066157 to K.E.K.)

## Declaration of competing interest

The authors declare no competing interests.

## Data Availability

Data will be made available on request.

## References

[1] Quek J., Chan K.E., Wong Z.Y., Tan C., Tan B., Lim W.H., Tan D.J.H., Tang A.S.P., Tay P., Xiao J., Yong J.N., Zeng R.W., Chew N.W.S., Nah B., Kulkarni A., Siddiqui M.S., Dan Y.Y., Wong V.W., Sanyal A.J., Noureddin M., Muthiah M., Ng C.H. (2023). Global prevalence of non-alcoholic fatty liver disease and non-alcoholic steatohepatitis in the overweight and obese population: a systematic review and meta-analysis. Lancet Gastroenterol Hepatol.

[2] Loomba R., Friedman S.L., Shulman G.I. (2021). Mechanisms and disease consequences of nonalcoholic fatty liver disease. Cell.

[3] Fan X., Song Y., Zhao J. (2024). Evolving liver disease insights from NAFLD to MASLD. Trends Endocrinol. Metabol..

[4] Yang S.Q., Lin H.Z., Mandal A.K., Huang J., Diehl A.M. (2001). Disrupted signaling and inhibited regeneration in obese mice with fatty livers: implications for nonalcoholic fatty liver disease pathophysiology. Hepatology.

[5] Picard C., Lambotte L., Starkel P., Sempoux C., Saliez A., Van den Berge V., Horsmans Y. (2002). Steatosis is not sufficient to cause an impaired regenerative response after partial hepatectomy in rats. J. Hepatol..

[6] Sundari P.N., Wilfred G., Ramakrishna B. (1997). Does oxidative protein damage play a role in the pathogenesis of carbon tetrachloride-induced liver injury in the rat?. Biochim. Biophys. Acta.

[7] Saxena N.K., Ikeda K., Rockey D.C., Friedman S.L., Anania F.A. (2002). Leptin in hepatic fibrosis: evidence for increased collagen production in stellate cells and lean littermates of ob/ob mice. Hepatology.

[8] Ikejima K., Honda H., Yoshikawa M., Hirose M., Kitamura T., Takei Y., Sato N. (2001). Leptin augments inflammatory and profibrogenic responses in the murine liver induced by hepatotoxic chemicals. Hepatology.

[9] Koteish A., Mae Diehl A. (2002). Animal models of steatohepatitis. Best Pract. Res. Clin. Gastroenterol..

[10] Diehl A.M. (2005). Lessons from animal models of NASH. Hepatol. Res..

[11] Leclercq I.A., Field J., Farrell G.C. (2003). Leptin-specific mechanisms for impaired liver regeneration in ob/ob mice after toxic injury. Gastroenterology.

[12] Potter J.J., Rennie-Tankesley L., Mezey E. (2003). Influence of leptin in the development of hepatic fibrosis produced in mice by Schistosoma mansoni infection and by chronic carbon tetrachloride administration. J. Hepatol..

[13] Kim K.E., Lee J., Shin H.J., Jeong E.A., Jang H.M., Ahn Y.J., An H.S., Lee J.Y., Shin M.C., Kim S.K., Yoo W.G., Kim W.H., Roh G.S. (2023). Lipocalin-2 activates hepatic stellate cells and promotes nonalcoholic steatohepatitis in high-fat diet-fed Ob/Ob mice. Hepatology.

[14] Kleiner D.E., Brunt E.M., Van Natta M., Behling C., Contos M.J., Cummings O.W., Ferrell L.D., Liu Y.C., Torbenson M.S., Unalp-Arida A., Yeh M., McCullough A.J., Sanyal A.J. (2005). N. Nonalcoholic Steatohepatitis Clinical Research, Design and validation of a histological scoring system for nonalcoholic fatty liver disease. Hepatology.

[15] Zhou Z., Xu M.J., Cai Y., Wang W., Jiang J.X., Varga Z.V., Feng D., Pacher P., Kunos G., Torok N.J., Gao B. (2018). Neutrophil-hepatic stellate cell interactions promote fibrosis in experimental steatohepatitis. Cell Mol Gastroenterol Hepatol.

[16] Brix A.E., Elgavish A., Nagy T.R., Gower B.A., Rhead W.J., Wood P.A. (2002). Evaluation of liver fatty acid oxidation in the leptin-deficient obese mouse. Mol. Genet. Metabol..

[17] Wortham M., He L., Gyamfi M., Copple B.L., Wan Y.J. (2008). The transition from fatty liver to NASH associates with SAMe depletion in db/db mice fed a methionine choline-deficient diet. Dig. Dis. Sci..

[18] Donnelly K.L., Smith C.I., Schwarzenberg S.J., Jessurun J., Boldt M.D., Parks E.J. (2005). Sources of fatty acids stored in liver and secreted via lipoproteins in patients with nonalcoholic fatty liver disease. J. Clin. Invest..

[19] Lambert J.E., Ramos-Roman M.A., Browning J.D., Parks E.J. (2014). Increased de novo lipogenesis is a distinct characteristic of individuals with nonalcoholic fatty liver disease. Gastroenterology.

[20] Heeboll S., Poulsen M.K., Ornstrup M.J., Kjaer T.N., Pedersen S.B., Nielsen S., Gronbaek H., Handberg A. (2017). Circulating sCD36 levels in patients with non-alcoholic fatty liver disease and controls. Int. J. Obes..

[21] Zeng H., Qin H., Liao M., Zheng E., Luo X., Xiao A., Li Y., Chen L., Wei L., Zhao L., Ruan X.Z., Yang P., Chen Y. (2022). CD36 promotes de novo lipogenesis in hepatocytes through INSIG2-dependent SREBP1 processing. Mol. Metabol..

[22] Nassir F., Adewole O.L., Brunt E.M., Abumrad N.A. (2013). CD36 deletion reduces VLDL secretion, modulates liver prostaglandins, and exacerbates hepatic steatosis in ob/ob mice. J. Lipid Res..

[23] Niu L., Cui X., Qi Y., Xie D., Wu Q., Chen X., Ge J., Liu Z. (2016). Involvement of TGF-beta1/smad3 signaling in carbon tetrachloride-induced acute liver injury in mice. PLoS One.

[24] Said M.M., Azab S.S., Saeed N.M., El-Demerdash E. (2018). Antifibrotic mechanism of pinocembrin: impact on oxidative stress, inflammation and TGF-beta/smad inhibition in rats. Ann. Hepatol..

[25] Krishnan A., Li X., Kao W.Y., Viker K., Butters K., Masuoka H., Knudsen B., Gores G., Charlton M. (2012). Lumican, an extracellular matrix proteoglycan, is a novel requisite for hepatic fibrosis. Lab. Invest..

[26] Bai Y., Wang W., Wang L., Ma L., Zhai D., Wang F., Shi R., Liu C., Xu Q., Chen G., Lu Z. (2021). Obacunone attenuates liver fibrosis with enhancing anti-oxidant effects of GPx-4 and inhibition of EMT. Molecules.

[27] Kamble P.G., Pereira M.J., Sidibeh C.O., Amini S., Sundbom M., Borjesson J.L., Eriksson J.W. (2016). Lipocalin 2 produces insulin resistance and can be upregulated by glucocorticoids in human adipose tissue. Mol. Cell. Endocrinol..

[28] Mosialou I., Shikhel S., Luo N., Petropoulou P.I., Panitsas K., Bisikirska B., Rothman N.J., Tenta R., Cariou B., Wargny M., Sornay-Rendu E., Nickolas T., Rubin M., Confavreux C.B., Kousteni S. (2020). Lipocalin-2 counteracts metabolic dysregulation in obesity and diabetes. J. Exp. Med..

[29] Stejskal D., Karpisek M., Humenanska V., Hanulova Z., Stejskal P., Kusnierova P., Petzel M. (2008). Lipocalin-2: development, analytical characterization, and clinical testing of a new ELISA. Horm. Metab. Res..

[30] Borkham-Kamphorst E., van de Leur E., Zimmermann H.W., Karlmark K.R., Tihaa L., Haas U., Tacke F., Berger T., Mak T.W., Weiskirchen R. (2013). Protective effects of lipocalin-2 (LCN2) in acute liver injury suggest a novel function in liver homeostasis. Biochim. Biophys. Acta.

[31] Duval D.L., Howard D., McCalden T.A., Billings R.E. (1990). The determination of myeloperoxidase activity in liver. Life Sci..

[32] Ohta Y., Imai Y., Matsura T., Kitagawa A., Yamada K. (2006). Preventive effect of neutropenia on carbon tetrachloride-induced hepatotoxicity in rats. J. Appl. Toxicol..

[33] Berres M.L., Koenen R.R., Rueland A., Zaldivar M.M., Heinrichs D., Sahin H., Schmitz P., Streetz K.L., Berg T., Gassler N., Weiskirchen R., Proudfoot A., Weber C., Trautwein C., Wasmuth H.E. (2010). Antagonism of the chemokine Ccl5 ameliorates experimental liver fibrosis in mice. J. Clin. Invest..

[34] Fan X., Lin L., Cui B., Zhao T., Mao L., Song Y., Wang X., Feng H., Qingxiang Y., Zhang J., Jiang K., Cao X., Wang B., Sun C. (2020). Therapeutic potential of genipin in various acute liver injury, fulminant hepatitis, NAFLD and other non-cancer liver diseases: more friend than foe. Pharmacol. Res..

[35] Ghanim B.Y., Qinna N.A. (2022). Nrf2/ARE axis signalling in hepatocyte cellular death. Mol. Biol. Rep..

[36] Choi Y.K., Kim Y.M. (2022). Beneficial and detrimental roles of heme oxygenase-1 in the neurovascular system. Int. J. Mol. Sci..

[37] Malaguarnera L., Madeddu R., Palio E., Arena N., Malaguarnera M. (2005). Heme oxygenase-1 levels and oxidative stress-related parameters in non-alcoholic fatty liver disease patients. J. Hepatol..

[38] Han C., Liu Y., Dai R., Ismail N., Su W., Li B. (2020). Ferroptosis and its potential role in human diseases. Front. Pharmacol..

[39] Raffaele M., Carota G., Sferrazzo G., Licari M., Barbagallo I., Sorrenti V., Signorelli S.S., Vanella L. (2019). Inhibition of heme oxygenase antioxidant activity exacerbates hepatic steatosis and fibrosis in vitro. Antioxidants.

[40] Zhao T., Yu Z., Zhou L., Wang X., Hui Y., Mao L., Fan X., Wang B., Zhao X., Sun C. (2022). Regulating Nrf2-GPx4 axis by bicyclol can prevent ferroptosis in carbon tetrachloride-induced acute liver injury in mice. Cell Death Dis..

[41] Tan Y., Huang Y., Mei R., Mao F., Yang D., Liu J., Xu W., Qian H., Yan Y. (2022). HucMSC-derived exosomes delivered BECN1 induces ferroptosis of hepatic stellate cells via regulating the xCT/GPX4 axis. Cell Death Dis..

[42] Dixon S.J., Lemberg K.M., Lamprecht M.R., Skouta R., Zaitsev E.M., Gleason C.E., Patel D.N., Bauer A.J., Cantley A.M., Yang W.S., Morrison B., Stockwell B.R. (2012). Ferroptosis: an iron-dependent form of nonapoptotic cell death. Cell.

[43] Asimakopoulou A., Weiskirchen S., Weiskirchen R. (2016). Lipocalin 2 (LCN2) expression in hepatic malfunction and therapy. Front. Physiol..

[44] Antonenkov V.D., Grunau S., Ohlmeier S., Hiltunen J.K. (2010). Peroxisomes are oxidative organelles. Antioxidants Redox Signal..

[45] Walton P.A., Brees C., Lismont C., Apanasets O., Fransen M. (2017). The peroxisomal import receptor PEX5 functions as a stress sensor, retaining catalase in the cytosol in times of oxidative stress. Biochim. Biophys. Acta Mol. Cell Res..

[46] Nitta Y., Muraoka-Hirayama S., Sakurai K. (2020). Catalase is required for peroxisome maintenance during adipogenesis. Biochim. Biophys. Acta Mol. Cell Biol. Lipids.

[47] Jonas W., Schwerbel K., Zellner L., Jahnert M., Gottmann P., Schurmann A. (2022). Alterations of lipid profile in livers with impaired lipophagy. Int. J. Mol. Sci..

[48] Kim K.E., Shin H.J., Ju Y., Jung Y., An H.S., Lee S.J., Jeong E.A., Lee J., Hwang G.S., Roh G.S. (2023). Intermittent fasting attenuates metabolic-dysfunction-associated steatohepatitis by enhancing the hepatic autophagy-lysosome pathway. Nutrients.

