# Supplementary material for: Carbon tetrachloride does not promote hepatic fibrosis in ob/ob mice via downregulation of lipocalin-2 protein
Source: Redox Biol. 2025 Jan 16;80:103506. doi: 10.1016/j.redox.2025.103506 (PMC11787671; doi:10.1016/j.redox.2025.103506)
Supplement: Multimedia component 1 [file mmc1.docx]

**Table S1. List of RT-PCR primers**

| **Gene name** | | **Primer sequences (mouse)** |  |
| --- | --- | --- | --- |
| *Tnf-α* | | Forward 5’ CCAGACCCTCACACTCAGATC 3' | |
|  | | Reverse 5’ CACTTGGTGGTTTGCTACGAC 3' | |
| *Il-1β* | | Forward 5’ GATCCACACTCTCCAGCTGCA 3' | |
|  | | Reverse 5’ TACAAGGAGAGACAAGCAACGACA 3' | |
| *Il-6* | | Forward 5’ GGTTTGCCGAGTAGACCTCA 3' | |
|  | | Reverse 5’ GTGGCTAAGGACCAAGACCA 3' | |
| *Mcp1* | | Forward 5’ CCACTCACCTGCTGCTACTCA 3' | |
|  | | Reverse 5’ TGGTGATCCTCTTGTAGCTCTCC 3' | |
| *F4/80* | | Forward 5’ GAGATTGTGGAAGCATCCGAGAC 3' | |
|  | | Reverse 5’ GATGACTGTACCCACATGGCTGA 3' | |
| *Cd68* | | Forward 5’ TGTCTGATCTTGCTAGGACCG 3' | |
|  | | Reverse 5’ GAGAGTAACGGCCTTTTTGTGA 3' | |
| *Tgf-β1* | | Forward 5’ TGGAGCAACATGTGGAACTC 3' | |
|  | | Reverse 5’ CAGCAGCCGGTTACCAAG 3' | |
| *Il-10* | | Forward 5’ CCAGGGAGATCCTTTGATGA 3' | |
|  | | Reverse 5’ AACTGGCCACAGTTTTCAGG 3' | |
| *Cxcl10* | | Forward 5’ GCTGCAACTGCATCCATATC 3' | |
|  | | Reverse 5’ AACACGTGGGCAGGATAG 3' | |
| *Lcn2* | | Forward 5’ CCAGTTCGCCATGGTATTTT 3' | |
|  |  | Reverse 5’ GGTGGGGACAGAGAAGATGA 3' | |
| *Gapdh* | | Forward 5’ AAATGGTGAAGGTCGGTGTG 3' | |
|  |  | Reverse 5’ CATGTAGTTGAGGTCAATGAAGG 3' | |

**Table S2. List of primary antibodies**

| **Antibody** | **Company** | **Catalog No.** | **Dilution(s)** | **Applications** | **Source** |
| --- | --- | --- | --- | --- | --- |
| CD36 | Novus | NB400 | 1:1000 | WB | Rabbit |
| Perilipin-2 | Abcam | ab52356 | 1:2000 | WB | Rabbit |
| PPAR-γ | Santa Cruz | sc-7196 | 1:1000 | WB | Rabbit |
| FAS | Cell signaling | #3189 | 1:1,000 | WB | Rabbit |
| SCD1 | Cell signaling | #2438 | 1:1,000 | WB | Rabbit |
| α-SMA | Sigma | A5228 | 1:1000 | WB | Mouse |
| Lumican | Abcam | ab168348 | 1:1000 | WB | Rabbit |
| Vimentin | Santa Cruz | sc-373717 | 1:1,000 | WB | Mouse |
| TGF-β1 | Santa Cruz | sc-146 | 1:1,000 | WB | Rabbit |
| LCN2 | R&D | AF1857 | 1:1000, 1:200 | WB, IF, IHC | Goat |
| pSTAT3 | Cell signaling | #9145 | 1:1,000 | WB | Rabbit |
| STAT3 | Cell signaling | #9139 | 1:1,000 | WB | Mouse |
| MMP9 | Abcam | ab38898 | 1:1,000 | WB | Rabbit |
| MPO | Abclonal | A1374 | 1:1,000, 1:200 | WB, IF | Rabbit |
| F4/80 | Santa Cruz | sc-377009 | 1:100 | IF | Mouse |
| NF-κBp65 | Cell signaling | #6956 | 1:1,000 | WB | Mouse |
| GS | Santa Cruz | sc-74430 | 1:5000, 1:200 | WB, IF | Mouse |
| Cleaved caspase-3 | Cell signaling | #9664 | 1:1,000 | WB | Rabbit |
| Nrf2 | Abcam | ab62352 | 1:1,000 | WB | Rabbit |
| NQO-1 | Abcam | ab34173 | 1:1,000 | WB | Rabbit |
| Catalase | Abcam | ab209211 | 1:1000, 1:200 | WB, IF | Rabbit |
| GPX-4 | Santa Cruz | sc-166570 | 1:1,000 | WB | Mouse |
| HO-1 | Enzo | ADI-SPA-895 | 1:1,000 | WB | Rabbit |
| 4-HNE | Abcam | ab46545 | 1:1,000 | WB | Rabbit |
| iNOS | DB Biotech | DB 003 | 1:1,000 | WB | Rabbit |
| LC3B | Cell signaling | #2775 | 1:1,000 | WB | Rabbit |
| p62 | Sigma | P0067 | 1:1000 | WB | Rabbit |
| p84 | Abcam | Ab487 | 1:3,000 | WB | Mouse |
| α-tubulin | Sigma | T5168 | 1:1,000 | WB | Mouse |
| β-actin | Sigma | A5441 | 1:1,000 | WB | Mouse |

WB, western blot; IF, immunofluorescence; IHC, immunohistochemistry
