# Supplementary material for: Carbon tetrachloride does not promote hepatic fibrosis in ob/ob mice via downregulation of lipocalin-2 protein
Source: Redox Biol. 2025 Jan 16;80:103506. doi: 10.1016/j.redox.2025.103506 (PMC11787671; doi:10.1016/j.redox.2025.103506)
Supplement: Multimedia component 2 [file mmc2.docx]

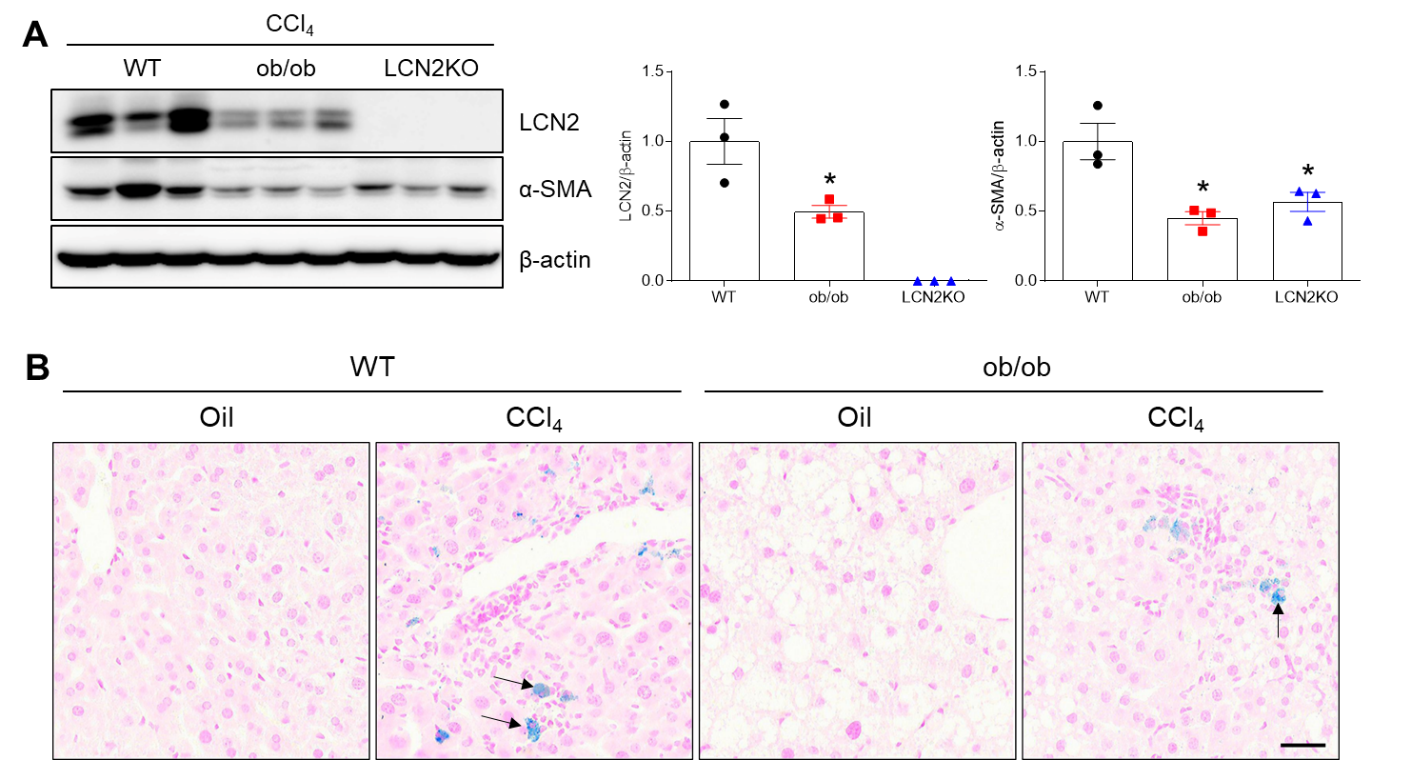


**Supplementary Figure 1. Effects of CCl_4_ treatment on hepatic fibrosis and iron accumulation. (A)** Western blotting and quantification of hepatic LCN2 and α-SMA protein levels in liver tissues of CCl_4_-treated WT, ob/ob, and LCN2KO mice. β-actin served as a loading control. (*n* = 3). Significance is determined using one-way ANOVA. *P < 0.05 vs. CCl_4_-treated WT mice. **(B)** Representative images of Perls Prussian blue staining in liver sections of WT and ob/ob mice with oil or CCl_4_. Arrows indicate iron-stained hepatocytes. Scale bar, 30 μm.
